# Effect of RARC-ERAS nursing program on clinical outcomes in patients undergoing RARC surgery: a retrospective, propensity matching study

**DOI:** 10.1007/s11701-024-01931-9

**Published:** 2024-04-10

**Authors:** Mang-mang He, Zhen-feng Zhou, Xiao-fen Yu, Chun-cong Zhou

**Affiliations:** 1 Department of the Operating Room, Zhejiang Provincial People’s Hospital, Affiliated People’s Hospital, Hangzhou Medical College, Hangzhou, 315000 Zhejiang China; 2 Department of Anesthesiology, Hangzhou Women’s Hospital (Hangzhou Maternity and Child Health Care Hospital, Hangzhou First People’s Hospital Qianjiang New City Campus, Zhejiang Chinese Medical University), Hangzhou, 310008 China; 3 Department of Urolithiasis and Anorectal Surgery, Ningbo No. 2 Hospital, 41 Xibei Street, Ningbo, 315010 Zhejiang China

**Keywords:** Enhanced recovery after surgery (ERAS), Radical cystectomy, Robot-assisted (RA), Bladder cancer, Deep vein thrombosis, Nursing

## Abstract

Currently, there is no specific perioperative nursing standard for RARC based on the ERAS concept. This retrospective study investigates to analyze the effect of RARC-ERAS nursing program on VTE and other clinical outcomes in patients undergoing RARC surgery. This retrospective study included 216 patients undergoing RARC surgery From January 1, 2022 to December 30, 2023, and propensity score adjustment analysis was applied. The study compares a control group receiving traditional nursing and an observation group receiving RARC-ERAS nursing program. Perioperative variables and other postoperative complications were retrieved from the hospital medical records. After propensity score matching, there were no significant differences in the demographic and clinical characteristics between the two groups (*p* > 0.05). The ERAS group exhibited aa significantly higher rate of postoperative unobstructed venous blood flow in the lower extremities by color Doppler ultrasound as compared to the control group (94.6% VS 80.4%, *p* = 0.042). Before anesthesia induction, lower preoperative anxiety and surgical information needs scores were observed in the ERAS group than in the control group (*p* < 0.05). Compared to the control group, the ERAS group demonstrated a shorter surgical duration, a lower incidence of perioperative hypothermia, less time needed for getting out of bed, anal exhaust, and for defecation after returning to the ward (*p* < 0.05). RARC-ERAS nursing program significantly increased the rate of postoperative unobstructed venous blood flow in the lower extremities by color doppler ultrasound, lower preoperative anxiety and intraoperative hypothermia in patients undergoing RARC. This nursing approach presents a valuable strategy for enhancing patient outcomes and merits further exploration in clinical practice.

*Trial registration:*ChiCTR2400081118; http://www.chictr.org.cn, Principal investigator: Mang-mang He, Date of registration: Feb 22, 2024.

**Supplementary Information:**

The online version contains supplementary material available at 10.1007/s11701-024-01931-9.

## Background

Venous thromboembolism (VTE) is strongly associated with cancer and is the second leading cause of death in cancer patients [1]. Patients undergoing robot-assisted radical cystectomy (RARC) for bladder cancer are at higher risk for VTE compared with other urological procedures [1]. Many enhanced recovery after surgery(ERAS) components have good data to support usage in patients undergoing RARC [2]. Most ERAS programs include multidisciplinary teams carrying out multimodal pathways to hasten recovery after a major operation.

The unique advantages of robotic technology provide a solid foundation for the development of RARC based on ERAS-related surgical nursing techniques [3]. ERAS nursing, which involves evidence-based approaches throughout the perioperative period, has proved to significantly reduce complications and hospital stays in robot-assisted surgeries [4]. Nurses play a pivotal role as direct operators in the execution of ERAS plans [5], are integral to the success of ERAS-based perioperative nursing. The operation of RARC surgery is complex and takes a long time. Currently, there is no specific perioperative nursing standard for RARC based on the ERAS concept.

After January 1, 2023, our hospital will apply the ERAS concept to the care of RARC surgery, optimize surgical care and implementation rules, and develop a RARC-ERAS nursing program based on the ERAS concept. We performed this retrospective study to analyze the effect of RARC-ERAS nursing program on VTE and other clinical outcomes in patients undergoing RARC surgery.

## Materials and methods

### Study population and design

After obtaining approval from the Ethics Committee of Zhejiang Provincial People's Hospital (Approval NO: 2023-217), 216 consecutive adult patients who underwent RARC were retrospectively collected from January 1, 2022 to December 30, 2023 in the hospital. We established based on the ERAS concept nursing measures since January 1, 2023. Therefore, a traditional group without the ERAS concept was identified as the control group from January 1, 2022 to December 31, 2022 and the ERAS group was identified as the observation group From January 1, 2023 to December 31, 2023.

All the patients conformed to the following conditions: 1) Patients diagnosed with invasive bladder urothelial carcinoma based on preoperative imaging, cystoscopy biopsy, or diagnostic bladder electroresection, with no evidence of systemic organ metastasis; 2) undergoing complete robot-assisted STAPLER method radical cystectomy, standard pelvic lymph node dissection, and in situ ileal U-shaped neobladder construction and all surgical procedures performed using the robot-assisted surgical system; 3) Absence of a history of external radiation therapy or intravenous chemotherapy before surgery, and no history of traditional open abdominal surgery; 4) Normal body temperature within the last 3 days and upon admission; 5) and color doppler ultrasound confirming unobstructed venous blood flow in the lower extremities before anesthesia.

Exclusion criteria comprised: 1) Intraoperative change of surgical approach to laparoscopy or open surgery; 2) History of VTE and prior anticoagulant or thrombolytic therapy before surgery; 3) Cardiovascular conditions prone to thrombosis, such as atrial fibrillation, congestive heart failure, pulmonary edema, etc.; 4) Disorders related to temperature regulation and metabolism; 5) Abnormalities in the local condition of the lower limbs, including swelling, gangrene, dermatitis, prior skin graft surgery, severe lower limb deformities, and ischemic vascular diseases like arterial sclerosis; 6) Transfusion of blood products during surgery; 7) Incomplete surgical data.

### Traditional nursing model

(1) Firstly, one day before surgery, the circulating nurse adhered to the preoperative visit protocol of the department in line with the surgical notice. (2) On the day of surgery, the circulating nurse played a crucial role in escorting the patient to the operating room. They confirmed the anesthesia method and coordinated with the surgeon to set up the operating room layout, following procedures analogous to conventional surgeries of the same type. (3) Once successful anesthesia was achieved, the patient was positioned according to the principles of the surgical position. (4) During the surgery, the circulating nurse took measures to enhance patient care. (5) At the conclusion of surgery, besides following routine procedures for surgeries of the same type, the number of uses of robotic instruments was documented using a marking pen. Detail of the traditional nursing model was listed in S1 Supplemental Appendix 1.

### RARC-ERAS nursing program

Establishment of a Robot-Assisted Surgical Nursing Team Based on the ERAS Concept and detail was listed in S1 Supplemental Appendix 1.

On the basis of the traditional nursing model, we established a robot-assisted surgical nursing team aligned with the principles of ERAS. The team comprises a nurse manager responsible for overseeing robot-assisted surgeries and five nurses specialized in robot-assisted surgery. One nurse serves as the coordinator of RARC-ERAS nursing program.

Responsibilities of nursing coordinator: (1) Surgical Progress Management: Coordinate pickup and transfer times for each surgical patient based on the pre-arranged surgical plan for the day before and the current day's progress. Minimize patient waiting times in the operating room to avoid prolonged waits and potentially extended anesthesia periods. (2) Robot-Assisted Surgical System Maintenance and Management: Ensure the accuracy of robot-assisted surgical system operation. Manage inventory of supplies and consumables. (3) Clinical Support: Assist in patient surgical preparation processes. Supervise nurses' technical skills during surgery. Provide help and advice for any issues that arise during surgical collaboration. (4) Education and Training: Conduct continuous training for the robot-assisted surgical nursing team in ERAS principles and specialized skills. Include training on preoperative visit content, processes, communication techniques, guidance for completing survey questionnaires, and methods for collecting evaluation data. Ensure the team's core competencies and job capabilities align with the evolving development of robot-assisted surgical techniques. (5) Quality Control: Communicate with the head of the robot-assisted surgery department, anesthesiology department, disinfection supply center, and engineers every 4 weeks to provide updates on the latest developments in robot-assisted surgery. Hold a quality control meeting after every 10 completed surgeries to discuss issues encountered during surgical collaboration, including any technical malfunctions during surgery. Conduct root cause analysis and sharing of corrective measures. Robot nurses should promptly report any issues discovered during their clinical practice to the coordinator while carrying out their routine surgical nursing duties.

### Data collection

Perioperative variables including demographic characteristic, individual history, preexisting risk factors, preoperative medications, co-morbidities, intraoperative data and other postoperative complications were retrieved from the hospital medical records, which occurred during the hospitalization, including the entire postoperative period up to discharge even if > 30 days. Laboratory test and part of preoperative characteristics were automated deriving from a computer database. Researchers were trained before data collection and an independent investigator reviewed all data. The researchers collecting postoperative endpoints were blinded to preoperative and intraoperative characteristics.

### Outcome definition

The primary outcome was the rate of unobstructed venous blood flow in the lower extremities by color doppler ultrasound during the postoperative period. Vascular conditions by color doppler ultrasound were assessed and reported as patency (unobstructed venous blood flow), stasis, and deep vein thrombosis (DVT) by an independent sonographer [6].

Other end points included in-hospital mortality and morbidities, duration of intensive care unit (ICU) stay, length of hospital stay (LOS) and duration of mechanical ventilation. Hospital morbidities included the following parts: (1) degree of preoperative anxiety score and surgical information demand score, were calculated according to the Amsterdam Preoperative Anxiety and Information Scale (APAIS) [7] at the first visit and before anesthesia induction. (2) perioperative hypothermia, defined as a core temperature dropping below 36 °C at any point from the beginning of the operation to leaving the Post-Anesthesia Care Unit (PACU) [8]. (3) the first time of getting out of bed, the first time of anal discharge, the first time of defecation after returning to the ward. (4) Postoperative pain using the numerical rating scale (NRS) [9].

### Statistical methods

Descriptive analyses of variables were used to summarize data. The normal distributed variables were expressed as mean ± standard deviation (SD) and compared with student’s t-test. Abnormal continuous variables were expressed as median (interquartile range (IQR)) and evaluated with Mann–Whitney U-test. respectively. Chi-square or Fisher's exact test was used to compare proportions between the two groups. Missing continuous variables of baseline parameters were less than 10% and were replaced by the median in Table 1. All reported p values were two sided, and values of *p* < 0.05 were considered to be statistically significant. Statistical analysis was performed with SPSS version 25.

**Table 1:** Demographic and Clinical characteristics of the two study groups before and after propensity score matching

Preoperative Characteristics	Entire Sample		*p* value	Propensity-Matched Group		*p* value
	ERAS			ERAS		
	Yes (*n* = 59)	No (*n* = 79)		Yes (*n* = 56)	No (*n* = 56)	
Age,yr	66 ± 14	67 ± 14	0.671	67 ± 14	67 ± 15	0.916
Male/female, no.(%)	50/9(84.7%)	60/19(75.9%)	0.204	47/9(83.9%)	48/8(85.7%)	0.792
BMI,kg/m^2^	21.4 ± 2.9	22.6 ± 3.0	0.022	21.6 ± 2.8	21.9 ± 2.6	0.495
ASA, no. (%)			0.127			0.118
I	1(1.7%)	3(3.8)		1(1.8%)	3(5.4%)	
II	40(67.8%)	55(69.6%)		38(67.9%)	37(66.1%)	
III	18(30.5%)	16(20.3%)		17(30.4%)	12(21.4%)	
IV	0(0%)	5(6.3%)		0(0%)	4(7.1%)	
Coexistent disease						
Hypertension, no. (%)	9(15.3%)	16(20.3%)	0.451	8(14.3%)	12(21.4%)	0.324
Diabetes, no. (%)	1(1.7%)	4(5.1%)	0.295	1(1.8%)	2(3.6%)	0.558
Cerebrovascular disease, no. (%)	3(5.1%)	3(3.8%)	0.714	2(3.6%)	2(3.6%)	> 0.999
Preoperative laboratory examination						
WBC, 10^9^	7.2 ± 3.2	6.7 ± 2.7	0.295	7.3 ± 3.4	6.8 ± 2.7	0.409
HCT,%	39 ± 7	37 ± 6	0.331	38 ± 7	37 ± 6	0.416
Cr,umol/L	99 ± 34	107 ± 90	0.504	99 ± 35	108 ± 79	0.435
Glucose level, mol/L	6.0 ± 1.6	6.3 ± 2.0	0.342	6.0 ± 1.6	6.2 ± 1.8	0.514
Pre-operative Characteristics						
Patient temperature on arrival in the operating room,℃	36.7 ± 0.4	36.7 ± 0.4	0.245	36.7 ± 0.4	36.7 ± 0.4	0.577
Anxiety score	14 ± 4	13 ± 3	0.980	8.1 ± 1.9	13.2 ± 3.4	< 0.001
Information desire score	7.0 ± 2.1	7.1 ± 1.9	0.732	4.6 ± 1.4	7.2 ± 1.8	< 0.001
Propensity score	0.48 ± 0.15	0.39 ± 0.14	< 0.001	0.46 ± 0.14	0.43 ± 0.12	0.176

# Fisher’s exact test was used;

*BMI* = body mass index; *ASA* = American Society of Anesthesiologists; *Hct* = Hematocrit

To minimize the effect of selection bias on outcomes, we used propensity score matching for clinical characteristics to reduce distortion by confounding factors. Using propensity score analysis by the method of nearest-neighbor matching, we generated a set of matched cases (ERAS) and controls (non-ERAS). A propensity score was generated for each patient from a multivariable logistic regression model on the basis of the covariates using clinical characteristics data (Table 1) from the institutional registry as independent variables, with treatment type (ERAS vs. non-ERAS) as a binary dependent variable. We matched patients using a greedy-matching algorithm with a caliper width of 0.1 of the estimated propensity score. A matching ratio of 1:1 was used. The inclusion matching indicators between the two groups included: gender, age, insulin dosage, BMI, ASA, Diabetes, and HCT value. We evaluated post-match covariate balance by comparing the balance of baseline covariates between patients with ERAS and non-ERAS before and after matching using absolute standardized differences [10].

## Results

### Baseline parameters

**Fig. 1:**
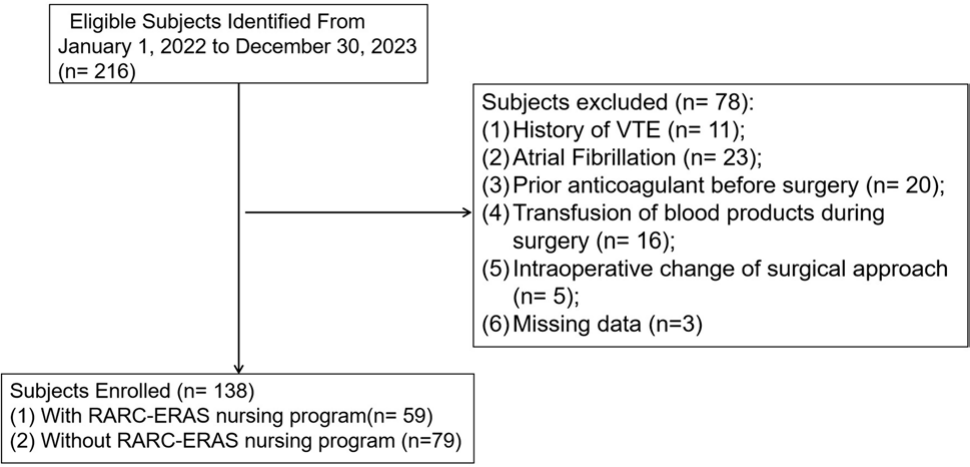
Study population recruitment summary. VTE = venous thromboembolism; RARC = robot-assisted laparoscopic radical cystectomy; ERAS = enhanced recovery after surgery

**Fig. 2:**
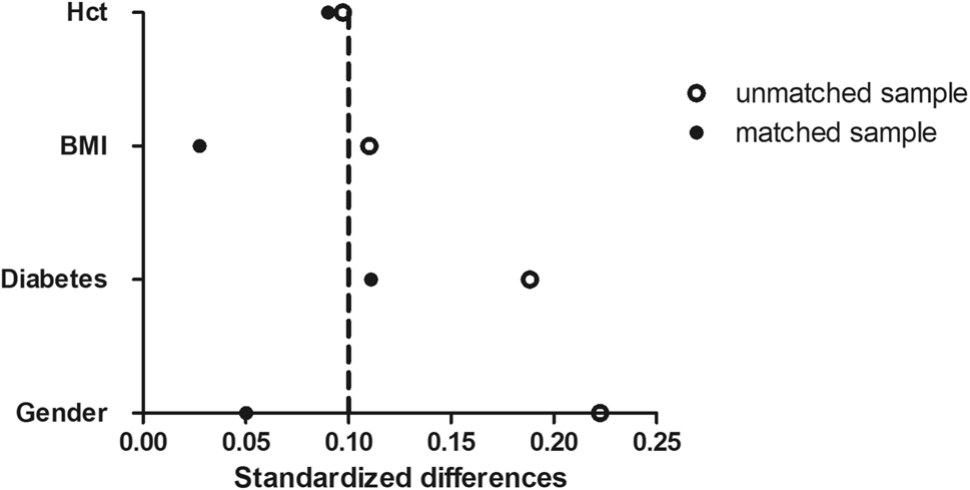
Absolute standardized differences in covariates between patients with RARC-ERAS nursing program and Not-RARC-ERAS nursing program group before and after PS matching. BMI = body mass index, Hct = Hematocrit

A total of 216 patients were identified and divided into two groups: patients who received RARC-ERAS nursing program (ERAS group, *n* = 59, 42.8%) and those who did not receive RARC-ERAS nursing program (non-ERAS group, *n* = 79, 57.2%) during the study period (Fig. 1). Compared with those in the control group, the BMI were lower and the propensity score was higher before matching in the ERAS group than that in the control group (*p* < 0.05). After matching, there were no significant differences in the general data between the two groups (*p* > 0.05, Table 1). According to the standardized difference, the covariate balance between the matched pairs was confirmed (Fig. 2).

### Perioperative outcomes after propensity matching

No case of deep vein thrombosis (DVT) was observed under color Doppler ultrasound examination during the postoperative period. The ERAS group exhibited a significantly higher rate of unobstructed venous blood flow in the lower extremities by color doppler ultrasound during the postoperative period as compared to the control group (94.6% VS 80.4%, *p* = 0.042, Table 2).

**Table 2:** Perioperative outcomes after propensity score matching

Outcomes	Propensity-matched		*p* valve
	ERAS		
	Yes (*n* = 56)	No (*n* = 56)	
Perioperative hypothermia	27(48.2%)	41(73.2%)	0.007
Duration of anesthesia [mean(SD); min]	222 ± 91	256 ± 82	0.040
Duration of surgery [mean(SD); min]	202 ± 89	234 ± 81	0.048
Introperative input [mean(SD); mL]	1013 ± 364	1154 ± 436	0.066
Venous blood flow in the lower extremities by color doppler ultrasound			0.042^#^
patency	53(94.6%)	45(80.4%)	
stasis	3(5.4%)	11(19.6%)	
DVT	0(0%)	0(0%)	
The first time to getting out of bed	15.5 ± 2.1	26.0 ± 3.6	< 0.001
The first time of anal exhaust	21 ± 4	28 ± 4	< 0.001
The first time of defecation	67 ± 20	81 ± 18	< 0.001
LOS [mean(SD); days]	14.5 ± 1.9	18.0 ± 2.6	< 0.001

*DVT* = deep vein thrombosis; *LOS* = length of hospital stay

^#^Fisher’s exact test was used

Before anesthesia induction, lower preoperative anxiety and surgical information needs scores were observed in the ERAS group than in the control group (*p* < 0.05, Table 1). Compared to the control group, the ERAS group demonstrated a shorter surgical duration, a lower incidence of perioperative hypothermia, less time needed for the first time to get out of bed, the first time of anal exhaust, and the first time of defecation after returning to the ward (*p* < 0.05, Table 2).

## Discussion

ERAS is a dynamic management strategy grounded in evidence-based medicine [11]. This approach involves a collaborative effort by a multi-disciplinary team (MDT), incorporating clinical, anesthesia, nutrition, and nursing disciplines. This retrospective and propensity-matching study found that RARC-ERAS nursing program significantly reduces the rate of postoperative stasis by color doppler ultrasound, preoperative anxiety, and intraoperative hypothermia in patients undergoing RARC. It was the first time that we paid attention to the role of nursing in ERAS in patients undergoing RARC surgery.

Although no case of postoperative DVT was found, we noticed that RARC-ERAS nursing program significantly reduces the rate of postoperative stasis by color doppler ultrasound in patients undergoing RARC, which might reduce the rate of postoperative DVT. The genesis of DVT is intricately linked to three factors: venous stasis, venous wall damage, and a hypercoagulable state. The malignancy-induced hypercoagulability, older age, major pelvic surgery, and cisplatin-based chemotherapy may all contribute to the higher risk [12]. The nature of malignant tumor disease and the method of endoscopic surgery can cause hypercoagulable blood, and the duration and complexity of surgery are important factors affecting the risk of postoperative DVT [13]. Intermittent pneumatic compression devices applied in ERAS period emulate muscle contraction, enhancing blood flow in the femoral and popliteal veins and reducing venous stasis. The study's findings indicate that RARC-ERAS nursing program exhibited shorter surgical durations.

The integration of ERAS principles with animated videos for preoperative education proves beneficial in mitigating patient anxiety [14]. This study indicated that RARC-ERAS nursing program, exposed to this educational approach, demonstrated significantly lower anxiety levels and reduced demand for surgical information before anesthesia induction compared to the control group, with statistically significant differences. Several factors contribute to this outcome. Firstly, RARC represents a highly sophisticated surgical technique, often unfamiliar to patients, leading to a lack of understanding. For those with heightened information needs, insufficient addressing of these needs by healthcare providers may escalate anxiety and hinder a comprehensive understanding of the medical condition, surgical procedure, and anesthesia-related details [15]. The ERAS-based preoperative visit covers comprehensive information, including surgery details, procedures, anesthesia, preoperative preparations, operating room environment, potential complications, and other relevant content. This approach caters to the informational needs of adult patients undergoing surgery [15], mitigating stress caused by misconceptions and exaggerated perceptions of surgical and anesthesia risks. Aasa et al. [16] suggest that providing patients with sufficient and relevant information before surgery effectively reduces anxiety and fear levels. Moreover, the content of the preoperative visit video, highlighting the benefits of ERAS treatment, provided patients and their families with a clear visual representation of the significance of ERAS practices, enhancing their understanding and compliance with ERAS principles. This, in turn, reinforces the successful implementation of nursing practices guided by the ERAS approach in clinical settings.

In alignment with ERAS principles, robot-assisted surgical nursing has shown promise in reducing intraoperative complications. The study's findings indicate that RARC-ERAS nursing program exhibited shorter surgical durations and a lower incidence of intraoperative hypothermia compared to the traditional nursing model. This observed enhancement can be attributed to several factors. The introduction of an ERAS robot-assisted surgical nursing coordinator role facilitated effective communication and coordination across multiple departments involved in surgery, ensuring seamless integration and promoting the standardization of ERAS protocols and procedures. Ongoing training in ERAS principles and specialized skills, coupled with regular communication every four weeks with the head of the robot-assisted surgery department, supported continuous professional development for surgical nurses and ensured the monitoring and continual improvement of surgical nursing quality.

The study's findings indicate that RARC-ERAS nursing program low incidence of intraoperative hypothermia compared to the traditional nursing model. During the surgical procedure, a systematic approach to maintaining warmth is adopted, including warmed intravenous fluids, warmed irrigation solutions for abdominal lavage, and the use of warming blankets. Concurrently, continuous monitoring of core body temperature is employed to minimize temperature fluctuations and mitigate the risk of complications associated with hypothermia. These measures are integral components of intraoperative nursing guided by ERAS principles [17].

We found that RARC-ERAS nursing program demonstrated a shorter surgical duration, a lower incidence of perioperative hypothermia, less time needed for getting out of bed, anal exhaust, and for defecation after returning to the ward. Consistent with previous research, Robot surgical nursing based on the ERAS concept [18] is beneficial for postoperative intestinal function recovery, assists anesthesiologists in implementing GDFT (goal-directed fluid therapy) during surgery, avoids excessive fluid input, reduces intestinal edema, guides patients to get out of bed early, and effectively promotes intestinal peristalsis.

## Limitations

The limitations of this study include its single-center, retrospective design, a relatively small number of cases, and a lack of longer postoperative clinical outcomes follow-up. Further confirmation of our study findings necessitates larger-scale prospective clinical research.

## Conclusions

RARC-ERAS nursing program significantly reduces the rate of postoperative stasis by color doppler ultrasound, preoperative anxiety, and intraoperative hypothermia in patients undergoing RARC. This nursing approach presents a valuable strategy for enhancing patient outcomes and merits further exploration in clinical practice.

## Supplementary Information

Below is the link to the electronic supplementary material.

Supplementary file1 (DOCX 34 KB)

## Data Availability

The datasets used during the current study are available from the corresponding author upon reasonable request.

## Abbreviations

APAIS: Amsterdam preoperative anxiety and information scale
ASA: American society of anesthesiologists
BMI: Body mass index
DVT: Deep vein thrombosis
ERAS: Enhanced recovery after surgery
GDFT: Goal-directed fluid therapy
HCT: Hematocrit
ICU: Intensive care unit
LOS: Length of hospital stay
MDT: Multi-disciplinary team
NRS: Numerical rating scale
PACU: Post-anesthesia care UNIT
RARC: Robot-assisted laparoscopic radical cystectomy;
VTE: Venous thromboembolism
