# Supplementary material for: Effect of RARC-ERAS nursing program on clinical outcomes in patients undergoing RARC surgery: a retrospective, propensity matching study
Source: J Robot Surg. 2024 Apr 10;18(1):170. doi: 10.1007/s11701-024-01931-9 (PMC11006731; doi:10.1007/s11701-024-01931-9)
Supplement: Supplementary file 1 — Supplementary file1 (DOCX 34 KB) [file 11701_2024_1931_MOESM1_ESM.docx]

**Detail of traditional nursing model**

(1) Firstly, one day before surgery, the circulating nurse adhered to the preoperative visit protocol of the department in line with the surgical notice. The nurse provided verbal health education, instructed the patient to fast for 8-12 hours, and abstain from drinking for 4-6 hours. Moreover, the nurse introduced the robot-assisted surgical process along with its advantages, offering targeted psychological counseling. Additionally, the nurse verified the signing of surgical-related medical documents and communicated with the surgeon to clarify the surgical plan, key points, and specific equipment requirements. On the day of surgery, the circulating nurse played a crucial role in escorting the patient to the operating room. They confirmed the anesthesia method and coordinated with the surgeon to set up the operating room layout, following procedures analogous to conventional surgeries of the same type. Once successful anesthesia was achieved, the patient was positioned according to the principles of the surgical position, with the patient in the supine position and legs elevated. The circulating nurse, in collaboration with the scrub nurse, assisted in establishing a sterile barrier, positioning puncture points, and adjusting the patient's position based on experience to achieve the head-down and legs-up position. Additionally, the circulating nurse cooperated with the medical team to park the bedside robotic arm system. During the surgery, the circulating nurse took measures to enhance patient care, such as covering the patient with a blanket to reduce unnecessary exposure, using a warmed irrigation solution for insulation, monitoring the patient's condition, and promptly adding surgical instruments according to the progress of the surgery. Simultaneously, the scrub nurse meticulously prepared intraoperative instruments and consumables, replaced surgical instruments in an orderly manner, promptly reported the status of the robotic arm system to the lead surgeon, and ensured the avoidance of instrument collisions. At the conclusion of surgery, besides following routine procedures for surgeries of the same type, the number of uses of robotic instruments was documented using a marking pen.

(2) Prevention of Hypothermia: Upon the patient's entry into the operating room, initiate preventive measures against hypothermia. Place a disposable full-body inflatable blanket (3M Company model 63500) on the uppermost layer of the operating table. Connect the blanket interface to the Bair Hugger (3M Company) warming unit, setting the inflation temperature to 37°C and adjusting the fan speed to the high setting. Ensure at least 30 minutes of pre-warming before anesthesia. Properly cover the patient with a blanket and maintain a room temperature of 24-26°C, controlled by the central panel, at least 30 minutes before anesthesia and post-surgery. Adjust the room temperature during surgery based on the surgeon's preference while ensuring the central control panel maintains a temperature not lower than 22°C and a relative humidity of 40%-60%. Utilize an intravenous fluid warmer set to 37°C and warm distilled water (43-45°C) in the lavage container of the fluid warmer. Minimize unnecessary exposure during skin disinfection and sterile field establishment. Monitor the patient's core body temperature closely, adjusting the temperature of the warming blanket machine in real-time to prevent hypothermia while avoiding overheating.

(3) Prevention of Deep Vein Thrombosis (DVT): Instruct the patient to externally rotate both lower limbs. Apply appropriately sized intermittent pneumatic compression device leg sleeves from the ankle, wrapping upwards to the thigh, with the knee joint exposed. Fasten the sleeves with adhesive strips on the side of the leg, ensuring untwisted tubing. Adjust tension to allow finger insertion and set pressures for the ankle, calf, and thigh according to instrument instructions (45 mmHg, 35 mmHg, 30 mmHg respectively). Seek subjective feedback from the patient for comfort. Position the patient supine with legs apart and use a cushion between the leg board and the popliteal fossa to maintain the knee joint in a functional position. After surgery and patient regains consciousness, replace the intermittent pneumatic compression device with elastic stockings [17].

(4) Prevention and Management of Surgical Site Infections: Strictly adhere to clean-flow operating room protocols. Supervise the surgical team to follow aseptic techniques. Administer prophylactic antibiotics per clinical guidelines [18], timed correctly 30 to 60 minutes before skin incision. In cases exceeding 3 hours or blood loss over 1500ml, prompt reminders to physicians for additional antibiotic orders and proper execution.

(5) Mitigation of Stress Reactions from Catheter Irritation: Avoid routine gastric tube placement. Emphasize urinary catheter placement post-general anesthesia.

(6) Assist Anesthesiologists in Achieving Goal-Directed Fluid Therapy (GDFT): Accurately assess and document fluid balance during surgery. Assist in monitoring both invasive and non-invasive indicators to achieve GDFT, preventing inadequate or excessive fluid resuscitation.

(7) Minimize Surgery-Related Factors Prolonging Operation Time: Scrub nurses refine preoperative material preparations based on surgery type and surgeon preference. Circulating nurses reconfirm the accuracy of scrub nurses' supplies and equipment preparation. Adjust patient positioning angles based on robotic arm range and surgical exposure requirements. Employ the AirSeal Intelligent Gas Management System (AS-IFS2, SurgiQuest, FDA No. 2014-2544025) to stabilize intra-abdominal pressure and address robotic failure codes promptly. Scrub nurses, through video imaging platforms, monitor the surgical process, anatomical features, and tissue characteristics. Constant communication with the lead surgeon is maintained to prevent collisions between robotic arms. At surgery conclusion, use video imaging platform cues to accurately record remaining instrument lifespan and place the mobile platform adjacent to the patient's bed.

**Detail of RARC-ERAS nursing program**

**Preoperative visit**

The preoperative visit is led by the ERAS robot surgical care coordinator to produce an animated video of the preoperative visit. The content of the animated video adds preoperative visit content based on the ERAS concept on the basis of traditional preoperative visit content including real-life pictures of the operating room and post anesthesia care unit (PACU) environment, robots, insulation and intermittent inflation pressure pumps, as well as the surgical position and positioning process, Take compound polyethylene glycol electrolyte powder for catharsis one day before operation, take less than 200ml glucose water orally when fasting 6 hours before operation, no contraindications such as diabetes 2 hours before operation, take warm water orally when there is diabetes, abdominal pressure urination and pelvic floor muscle exercise method and significance, characteristics and advantages of robotic surgery, general situation of robotic surgery in our hospital, general surgical process of RARC, cooperation before tracheal tube removal and possible discomfort after extubation The possible postoperative discomfort symptoms include surgical stimulation, the disease itself, side effects caused by the use of analgesics, and corresponding prevention and treatment measures. The purpose and specific measures of ERAS implementation, as well as the benefits obtained by patients through ERAS treatment mode, are also included. The above pictures and videos are made into animated videos based on the story plot and accompanied by text explanations and explanations. The completed interview videos are stored on tablets and updated monthly, Optimize details and timely add the latest disciplinary updates on ERAS and robotic surgery; On the afternoon before surgery, the touring nurse who was responsible for the surgery brought a tablet computer with stored interview videos into the ward. Based on reviewing the patient's medical history, diagnosis, and various examinations, they went to the patient's bedside with the responsible nurse in the ward and used easy to understand language and animation videos to adopt appropriate communication skills according to the patient's lifestyle habits and personality characteristics provided by the responsible nurse. Personalized education was provided to the patient and their family members, And promptly answer any questions raised by the patient, complete the education and comprehensive evaluation of the patient's condition, including nutritional status, overall skin and blood vessel condition, maximum tolerance angle of joint movement, etc. At the same time, instruct the patient to bring medical elastic socks into the operating room on the surgical day; After the visit, copy the video content into the mobile nursing vehicle in the ward. Participate in preoperative discussions, prepare robotic and endoscopic instruments based on the surgeon's habits and anatomical characteristics of the surgical site, clarify the layout of the operating room and surgical position requirements, review the Caprini score^[1]^ for deep venous thrombosis (DVT) at the time of issuing the patient's surgical notice, and confirm the surgeon's prescription for the use of intermittent inflation and compression devices during surgery.

Intraoperative cooperation:

(1) Prevention of hypothermia: Patients enter the operating room and lie on the top layer, laying a disposable full body model 63500 3M company full body inflatable blanket. Its interface is connected to the operating bed of the Bair Hugger (3M company) heater, and the heater is turned on. The inflation temperature is set to 37 ℃, and the wind speed is set to fast speed. Ensure that at least 30 minutes of pre insulation is given before anesthesia, and the cotton quilt is properly covered, At least 30 minutes before anesthesia and at the end of surgery, the central control panel room temperature should be controlled at 24-26 ℃. During surgery, the subjective sensation of the surgeon should be taken into account to adjust the room temperature. However, the central control panel should not lower the room temperature than 22 ℃ and the relative humidity should be 40-60%. The intravenous infusion heater should be turned on to 37 ℃, and the distilled water for flushing the body cavity in a constant temperature box should be heated to 43-45 ℃. When disinfecting the skin and establishing a sterile area, unnecessary exposure should be minimized as much as possible, Strictly observe the patient's core body temperature, adjust the temperature of the temperature blanket machine in real time, and prevent hypothermia while avoiding high body temperature; (2) Prevention of DVT: Instruct the patient to abduct both lower limbs and wrap a suitable type of intermittent inflation pressure device leg cover from the ankle to the thigh according to the circumference of the patient's calf and thigh, exposing the knee joint. Then, sequentially attach the buckle placed on the side of the leg, connect the pipeline, confirm that the pipeline is not twisted or compressed, and the tightness should be suitable to accommodate one finger. Adjust the pressure on the ankle, calf, and thigh according to the instrument manual to 45mmHg, 35mmHg, and 30mmHg, respectively, Simultaneously inquire about the patient's subjective feelings until they feel comfortable; According to the principle of positioning and considering the patient's comfort, place the patient in a supine split leg position to avoid knee joint overextension. Place a cotton pad between the leg board and the popliteal fossa to keep the knee joint in a functional position. After the surgery, the patient should wake up and replace the intermittent inflation compression device with elastic socks; (3) Prevention and treatment of surgical site infections: Strictly follow the management standards of laminar flow clean operating rooms, supervise the surgical team to strictly implement aseptic operations, follow the clinical application guidelines of antibiotics [18], and correctly prevent the use of antibiotics according to medical advice 30 minutes to 60 minutes before skin cutting. If the surgical time exceeds 3 hours or the bleeding volume exceeds 1500ml, remind the doctor to issue additional antibiotic orders in a timely manner and execute them correctly; (4) Reduce the stress response caused by catheter stimulation in patients: unconventional placement of gastric tubes, emphasizing the placement of catheters after general anesthesia takes effect; (5) Assist anesthesiologists in achieving goal-directed fluid therapy (GDFT) during surgery: correctly evaluate and record inflow and outflow during surgery, assist anesthesiologists in observing invasive and non-invasive monitoring indicators to achieve GDFT, and avoid insufficient and overloaded volume; (6) Avoiding surgical nursing factors and prolonging surgical time: The instrument nurse continuously improves preoperative item preparation based on the type of surgery and the habits of the chief surgeon. Before the surgery begins, the touring nurse reconfirms the correctness of the instrument nurse's consumables and instrument preparation, and adjusts the body position angle according to the range of motion of the robotic arm and the exposure needs of the surgical department, During the operation, the AirSeal intelligent pneumoperitoneum system (AS-IFS2, SurgiQuest, USA, Food and Drug Administration (Jin) Zi 2014 No. 2544025) was used to stabilize pneumoperitoneum pressure, quickly and correctly identify and process robot fault codes. The instrument nurse paid attention to the surgical process, anatomical characteristics of the surgical site, and tissue properties through the video imaging platform, quickly replaced the instrument, and correctly selected different types of needles and sutures, and cut them to the appropriate length, At the same time, pay close attention to the aerial position of the instrument arm, communicate with the chief surgeon at any time, and avoid collision with the instrument arm. After the surgery, correctly record the remaining life of the instrument according to the video imaging platform prompts and fold the bedside moving platform to place it in a fixed position.

**References**

[1] Bahl V, Hu HM, Henke PK, Wakefield TW, Campbell DA Jr, Caprini JA. A validation study of a retrospective venous thromboembolism risk scoring method. Ann Surg. 2010. 251(2): 344-50.
